# Anticipatory Postural Control of Stability during Gait Initiation Over Obstacles of Different Height and Distance Made Under Reaction-Time and Self-Initiated Instructions

**DOI:** 10.3389/fnhum.2016.00449

**Published:** 2016-09-07

**Authors:** Eric Yiou, Romain Artico, Claudine A. Teyssedre, Ombeline Labaune, Paul Fourcade

**Affiliations:** ^1^CIAMS, Université Paris Sud, Université Paris-SaclayOrsay, France; ^2^CIAMS, Université d’OrléansOrléans, France

**Keywords:** stability, anticipatory postural adjustments, obstacle clearance, mechanical modeling, temporal pressure, gait initiation, motor coordination, human

## Abstract

Despite the abundant literature on obstacle crossing in humans, the question of how the central nervous system (CNS) controls postural stability during gait initiation with the goal to clear an obstacle remains unclear. Stabilizing features of gait initiation include anticipatory postural adjustments (APAs) and lateral swing foot placement. To answer the above question, 14 participants initiated gait as fast as possible in three conditions of obstacle height, three conditions of obstacle distance and one obstacle-free (control) condition. Each of these conditions was performed with two levels of temporal pressure: reaction-time (high-pressure) and self-initiated (low-pressure) movements. A mechanical model of the body falling laterally under the influence of gravity and submitted to an elastic restoring force is proposed to assess the effect of initial (foot-off) center-of-mass position and velocity (or “initial center-of-mass set”) on the stability at foot-contact. Results showed that the anticipatory peak of mediolateral (ML) center-of-pressure shift, the initial ML center-of-mass velocity and the duration of the swing phase, of gait initiation increased with obstacle height, but not with obstacle distance. These results suggest that ML APAs are scaled with swing duration in order to maintain an equivalent stability across experimental conditions. This statement is strengthened by the results obtained with the mechanical model, which showed how stability would be degraded if there was no adaptation of the initial center-of-mass set to swing duration. The anteroposterior (AP) component of APAs varied also according to obstacle height and distance, but in an opposite way to the ML component. Indeed, results showed that the anticipatory peak of backward center-of-pressure shift and the initial forward center-of-mass set decreased with obstacle height, probably in order to limit the risk to trip over the obstacle, while the forward center-of-mass velocity at foot-off increased with obstacle distance, allowing a further step to be taken. These effects of obstacle height and distance were globally similar under low and high-temporal pressure. Collectively, these findings imply that the CNS is able to predict the potential instability elicited by the obstacle clearance and that it scales the spatiotemporal parameters of APAs accordingly.

## Introduction

The control of postural stability is crucial for the efficient performance of day-to-day motor tasks. Like all terrestrial species, humans move around in a gravity field that permanently induces postural destabilization through its attracting effect towards the center of the earth. Major questions in motor control relate to the way in which humans are able to maintain stability during motor tasks that involve whole body progression, such as locomotor tasks, and how they adapt to environmental constraints, e.g., when clearing an obstacle. Gait initiation, which corresponds to the transient period between quiet standing and swing foot contact with the ground, is a classical paradigm for studying balance control mechanisms during complex whole body movement (e.g., Brenière et al., 1987; Lyon and Day, 1997, 2005; McIlroy and Maki, 1999; Yiou et al., 2012a for a recent review; Caderby et al., 2014). The act of lifting the swing foot from the ground to step in the desired direction does indeed induce a reduction in the size of the mediolateral (ML) base of support, moving from a bipedal to a unipedal stance. If the center of mass is not repositioned above (or closer to) the limits of the new base of support -i.e., the stance foot–, the body will topple towards the swing leg side during the single stance phase (or “swing phase”) of gait initiation under the effect of gravity, which may cause lateral instability at foot contact. This instability is invariably attenuated during volitional stepping by the development of dynamic postural phenomena that occur before the swing phase. These dynamic phenomena correspond to “anticipatory postural adjustments” (APAs). They include a center of pressure shift towards the swing leg side which serves to accelerate the center of mass in the opposite direction, i.e., towards the stance leg side (Do et al., 1991; Jian et al., 1993; McIlroy and Maki, 1999; Nouillot et al., 2000; Caderby et al., 2014; Yiou et al., 2016). If not enough APAs are generated in the ML direction, a strategy of base of support enlarging, associated with a more lateral swing foot placement, has been shown to be triggered to maintain stability (Zettel et al., 2002; Caderby et al., 2014). In addition to this putative stabilizing function, APAs have been shown to provide the dynamic conditions for whole body progression in the desired direction. For example, during gait initiation, APAs in the anteroposterior (AP) direction include a backwards center of pressure shift that promotes the forward propulsive forces necessary to reach the intended center of mass velocity and step length (Brenière et al., 1987; Lepers and Brenière, 1995; Michel and Chong, 2004).

Postural stability during gait initiation might be further challenged by the presence of an obstacle that needs to be cleared. There has been extensive literature on the control of obstacle crossing during ongoing locomotion, especially in regards to the role of vision (e.g., Mohagheghi et al., 2004; Patla and Greig, 2006; Marigold et al., 2007). In comparison, the question how the postural and the focal components of gait initiation over an obstacle are coordinated to ensure safe body progression has received much less attention (e.g., Brunt et al., 1999; Yiou et al., 2016). Yet, it is known that gait initiation is among the motor activities associated with the highest proportion of falls in the elderly (Robinovitch et al., 2013). In addition, the most frequent cause of falling in this population is an incorrect weight transfer, which, as stated above, is one of the major functions of APAs. In addition to the risk of tripping over the obstacle, the presence of an obstacle gives rise to an increase in the duration of the swing phase and therefore an increase in the potential for lateral instability (Zettel et al., 2002; Yiou et al., 2016). Hence, it is surprising that previous studies on the influence of an obstacle on the lateral motion of the center of mass during ongoing walking have reported that lateral stability remained unchanged when the height of the obstacle was varied (Chou et al., 2001; Hahn and Chou, 2004). This result led the authors to suggest the existence of some forms of adaptive postural mechanisms aimed at compensating for the increased potential instability related to obstacle height. However, these mechanisms remain to be clarified. To date, the question of whether or not the stabilizing mechanisms of gait initiation can accommodate obstacle constraints has been investigated in only one study (Yiou et al., 2016). This study showed that the amplitude of ML APAs was larger in the obstacle condition than in the obstacle free (control) condition. It was suggested that this increase was responsible for the maintenance of postural stability at swing foot contact. Similar results were obtained by Zettel et al. (2002) during their comparison of reactive stepping over an obstacle in response to a brisk plate-form shift with the same reactive stepping in an obstacle-free condition. However, these studies are all limited by the fact that only one obstacle height and distance were tested. Thus, one can question the generalizability of these results and more specifically, the extent to which the central nervous system (CNS) is able to adjust the stabilizing features of gait initiation (including ML APAs and base of support enlargement) to match changes in obstacle height and distance and the related potential for instability. Moreover, subjects of these studies invariably increased ML APAs when stepping (voluntarily or reactively) over the obstacle; thus, it could not be established that the absence of such an increase would have necessarily led to instability at foot contact. Lyon and Day (1997, 2005) used a single-segment mechanical model in which the body falls freely under the influence of gravity to predict the magnitude of the lateral center of mass fall during the swing phase of step initiation. In the present study, we elaborated on a mechanical model that was based on these last two studies in order to investigate how changes in the parameters of ML APAs can impact on postural stability at foot contact. Such modeling may thus provide further insight into the adaptability of the postural system to environmental constraints.

This study aims to investigate how the CNS controls postural stability during gait initiation when clearing obstacles of different heights and distances. Changes in obstacle height and distance were expected to bring about modulation of the swing phase and give rise to instability. In addition, as daily motor tasks may be performed under various temporal pressure constraints, gait initiation trials were performed in reaction-time (high pressure) and self-initiated (low pressure) conditions. Our previous study (Yiou et al., 2016) showed that the duration of APAs associated with gait initiation when faced with an obstacle was shorter under high pressure than under low pressure. This difference in duration was compensated by an increase in the amplitude of ML APAs. As only one obstacle height and distance were used (one 20 cm high obstacle, placed at a 20% body height distance from the participant), it can be questioned whether the CNS uses a similar anticipatory postural adaptation to temporal pressure when the obstacle constraints are manipulated. This is particularly the case when height and distance are increased, thereby placing a higher level of stress on the postural system.

This question might be addressed in regards to current theory on motor control, according to which our nervous system would possess neural structures (or internal models) that predict the future state of a system given the current state and the sensorimotor control signals (Wolpert and Flanagan, 2001). Such prediction would allow us to achieve rapid and accurate voluntary behavior despite the difficulties presented by motor noise, delayed sensory feedback, and a complex musculoskeletal apparatus. As stressed in Mille et al. (2012), it is clear that “the anticipatory nature of the APAs involves a role for motor prediction”. Specifically, APAs structure would reflect the existence of internal models that predicts the destabilizing effect associated with the stepping (Lyon and Day, 1997, 2005). When stepping over an obstacle, it can therefore be expected that APAs will be scaled according to the potential destabilization associated with obstacle constraints. We thus hypothesize that the stabilizing features of gait initiation are scaled according to the changes in the swing phase duration that is associated with obstacle height and/or distance. More specifically, it is expected that a greater swing phase duration will be associated with larger ML APAs and eventually, a larger base of support in order to maintain unchanged postural stability at swing foot contact. Similar effects of obstacle constraints are expected under low and high temporal pressure conditions. However, APAs of larger amplitude and lower duration are expected in the high pressure condition compared with the low pressure condition. Mechanical modeling of the whole body during gait initiation is expected to reveal the extent to which postural stability at foot contact may be degraded in case ML APAs are not adequately scaled to modifications in swing duration induced by obstacle constraints.

## Materials and Methods

### Participants

Fourteen subjects (eight males and six females, aged 23.2 ± 4 years [mean ± SD], height 173.4 ± 7.3 cm and weight 65.8 ± 8.7 kg) participated in the experiment. All were free of any known neuromuscular disorders. They gave written informed consent after being instructed as to the nature and purpose of the experiment, which was approved by the local ethics committee. The study conformed to the standards set by the Declaration of Helsinki.

### Experimental Protocol

**Figure 1:**
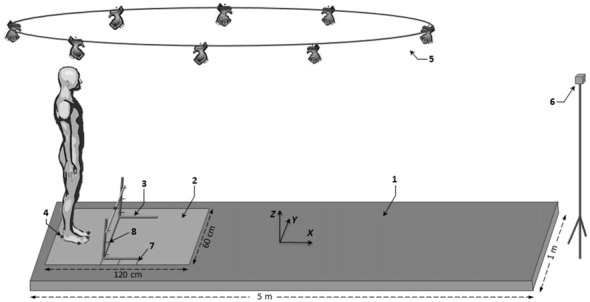
**Schematic illustration of the experimental set-up.** Key: (1) walkway; (2) force-plate; (3) obstacle; (4) reflective marker; (5) Vicon camera; (6) visual target; (7) obstacle distance marker; and (8) obstacle height marker.

Participants were requested to initiate gait as fast as possible with their preferred limb while clearing an obstacle placed in front of them (Figure 1). Three conditions of obstacle height (2.5%, 5% and 10% of each subject’s height), three conditions of obstacle distance (10%, 20% and 30% of each subject’s height) and an obstacle-free control condition were used. The three obstacle distances corresponded to 21%, 42% and 63% of the step length obtained in the control condition, respectively. Each condition of obstacle height and distance was realized in two blocks, which differed in terms of their level of temporal pressure constraint: a reaction-time and a self-initiated block. In the reaction-time block (high temporal pressure), participants were instructed to initiate gait “as soon as possible” after an acoustic signal was given. In the self-initiated block (low temporal pressure), they were instructed to initiate gait when they felt ready, after receiving an “all set” signal; it was made clear that the “all set” signal was not a “go” signal and that they could take as much time as they needed to prepare their movements. The order of conditions within one given block and the order of the blocks were randomized between participants. In each condition, subjects were allowed two familiarization trials. Five trials were then recorded. A 3-min rest was imposed between two successive conditions to avoid fatigue. In each condition, the participants initially stood upright with their feet hip-width apart, their arms hanging loosely either side of their body and their body weight evenly distributed between their legs. The boundaries of their feet in the initial posture were outlined on the force plate, and participants were instructed to systematically reposition their feet within these marks under supervision. They were repeatedly reminded of the task instructions.

### Materials

Gait was initiated on a force plate (600 × 1200 mm, AMTI, Watertown, MA, USA) located at the beginning of a five-meter track (Figure 1). The force plate was embedded in the track and was large enough to allow the participant’s swing foot to systematically land on it at the end of gait initiation. After crossing the obstacle, participants walked to the end of the track, then stood still for a few seconds before returning to their starting position. The obstacle consisted of a lightweight wooden rod (length: 65 cm; diameter: 1 cm) that rested on two adjustable upright standards. The participant’s toes served as the reference point for positioning the obstacle at the various distances. Reflective skin markers (9 mm in diameter) were placed bilaterally at the hallux (toe marker), head of the fifth metatarsal (metatarsal marker), posterior calcaneus (heel marker) and at the middle of the top of the obstacle (obstacle marker). A V8i VICON eight-camera (Mcam2) motion capture system (Oxford Metrics Ltd., UK) with 64 analog channels was used to record the movement of the foot markers and to detect the position of the obstacle. Kinematic and kinetic data were collected simultaneously at a rate of 500 Hz. Data acquisition and stimulus display were controlled by a custom-made program written in Matlab^TM^ (R2009b, The MathWorks Inc., Natick, MA, USA).

### Data Analysis

Kinematic and force plate data were low-pass filtered using a Butterworth filter with a 15 Hz (Mickelborough et al., 2000) and a 10 Hz (Caderby et al., 2014) cut-off frequency, respectively. The ML (*yP*) and AP (*xP*) coordinates of the center of pressure were computed from force plate data as follows:

(1) $$yP\;=\;\frac{Mx+Fy\times dz}{Fz}$$

(2) $$xP\;=\;\frac{-My+Fx\times dz}{Fz}$$

where *Mx* and *My* are the moments around the AP and ML axes, respectively; *Fy*, *Fx* and *Fz* are the ML, AP and vertical ground reaction forces, respectively; and *dz* is the distance between the surface of the force plate and its origin.

Instantaneous acceleration of the center of mass along the AP and ML axes was determined from the ground reaction force according to Newton’s second law. Center of mass velocity and displacement were computed by successive numerical integrations of center of mass acceleration using integration constants equal to zero, i.e., initial velocity and displacement null (Brenière et al., 1987). The following instants were determined from biomechanical traces: gait initiation onset (t_0_), swing heel off, swing toe off and swing foot contact. T_0_ and foot contact were determined from force plate data, whereas heel off and toe off were determined from VICON data. Two t_0_ times were estimated, one for the ML axis and one for the AP axis. The t_0_ times corresponded to the instants when the ML or AP center of pressure trace deviated 2.5 standard deviations from its baseline value. Heel off and toe off corresponded to the instants when the vertical position of the swing heel marker and the anterior position of the swing toe marker increased by 3 mm from their position in the initial static posture. Foot contact corresponded to the instant when the ML and AP center of pressure traces shifted abruptly laterally towards the swing leg side, and forwardly, respectively (this abrupt shift occurred at the same instant in the two traces).

### Mechanical Model

**Figure 2:**
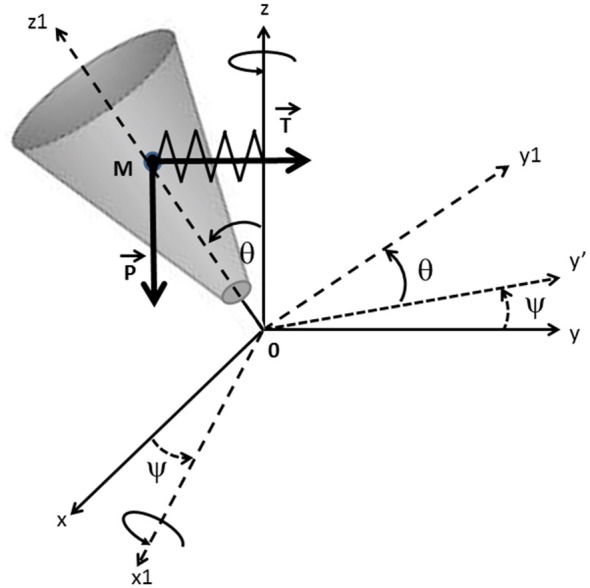
**Mechanical model.** The mechanical model is represented as a conic inverted pendulum which pivots about a fixed point 0. Body displacement during the swing phase (from toe off to foot contact) presents five degrees of freedom on the absolute referential (0; *x*; *y*; *z*). (0; *x*_1_; *y*_1_; *z*_1_) are the main axes of the inertia momentum of the solid body after precession ψ around *z* and nutation θ around *x*_1_. The center of mass m falls under the influence of the gravity force *P* and the elastic restoring force *T*. The initial position and velocity of the cone correspond to the position and velocity of the subject’s center of mass at toe off.

In the present study, the human body was modeled during the swing phase of gait initiation (from toe off to foot contact) as a single conic inverted pendulum which rotates about a fixed point 0 (Figure 2). This model was based on work carried out in earlier studies (Jian et al., 1993; MacKinnon and Winter, 1993; Lyon and Day, 1997, 2005). The displacement of this cone had five degrees of freedom on the absolute referential (0, *x*, *y*, *z*), i.e., three translations and two rotations. A new referential (0, *x*_1_, *y*_1_, *z*_1_) was considered after precession ψ around *z* and nutation θ around *x*_1_, in which the inertia momentum of the body was expressed with its eigenvalues (Winter et al., 1990). The proper rotation ψ around *z*_1_ with respect to ψ and θ was neglected. During the swing phase, we considered that the center of mass was falling laterally under the influence of two forces: the gravity force *P* = *mg* (where *m* is the mass of the solid, and *g* is the gravitational acceleration) and an elastic restoring force *T* that reflects active muscular control of the movement (Farley and Morgenroth, 1999; Morasso and Schieppati, 1999), with *T* = *k*|yM| (where *k* is the stiffness of the hip abductor muscles acting on the stance leg side during the swing phase (Winter, 1995) and *|yM|* is the absolute value of the ML center of mass shift, which was systematically oriented towards the swing leg side (positive values) during the swing phase). The initial position and velocity of the cone corresponded to the position and velocity of the subject’s center of mass at toe off. The addition of a restoring force on the conic model was necessary in order to control the initial velocity at toe off. Without this supplementary force, the conic pendulum would fall towards the stance leg side in most trials.

The equation of motion in (0, *x_1_*, *y*_1_, *z*_1_) was:

(3) $$OM\times \left(mg+k\left|\text{yM}\right|\right)\;=\;\frac{\text{d}\sigma_{/0}}{\text{d}t}$$

Where *OM* = *l*_M_ *z*_1_, with *l*_M_: distance of the center of mass along *z*_1_, and *σ/0*: angular momentum computed as *σ/0* = *I*_/0_ Ω, where *I*_/0_ is the diagonal matrix of inertia along the main axes (*x_1_, y_1_, z_1_*) and Ω is the total angular velocity:

(4) $$\Omega \;=\;\dot{\theta }x_{1}+\dot{\psi }\sin\theta y_{1}+\dot{\psi }\cos\theta z_{1}$$

Finally, the differential equations of angular movement were:

(5) $$\ddot{\theta }\;\text{​}=\text{​}\;\;\frac{mgl_{\text{M}}\sin\theta \text{​}+\text{​}k\left|yM\right|l_{\text{M}}\sin\psi \cos\theta \text{​}-\text{​}\overset{}{{\dot{\psi }}^{2}}\cos\theta \sin\theta \text{​}\left(I_{oz_{1}}\text{​}-\text{​}I_{ox_{1}}\right)\text{​}}{I_{ox_{1}}}$$

(6) $$\ddot{\psi }\;=\;\frac{k\left|yM\right|l_{\text{M}}\cos\psi \sin\theta -2\dot{\theta }\dot{\psi }\cos\theta \sin\theta \left(I_{ox_{1}}-I_{oz_{1}}\right)}{I_{ox_{1}}\sin^{2}\theta +I_{oz_{1}}\cos^{2}\theta }$$

The subsequent motion of the model’s center of mass was predicted by solving numerically the differential equations of motion using a fourth order Runge-Kutta algorithm. The spherical coordinates numerically computed (**l*_M_, θ, ψ*) were then transformed into Cartesian coordinates (*x, y, z*) in order to compare experimental data with the model’s theoretical data.

### Dependant Variables

#### Experimental Variables

**Figure 3:**
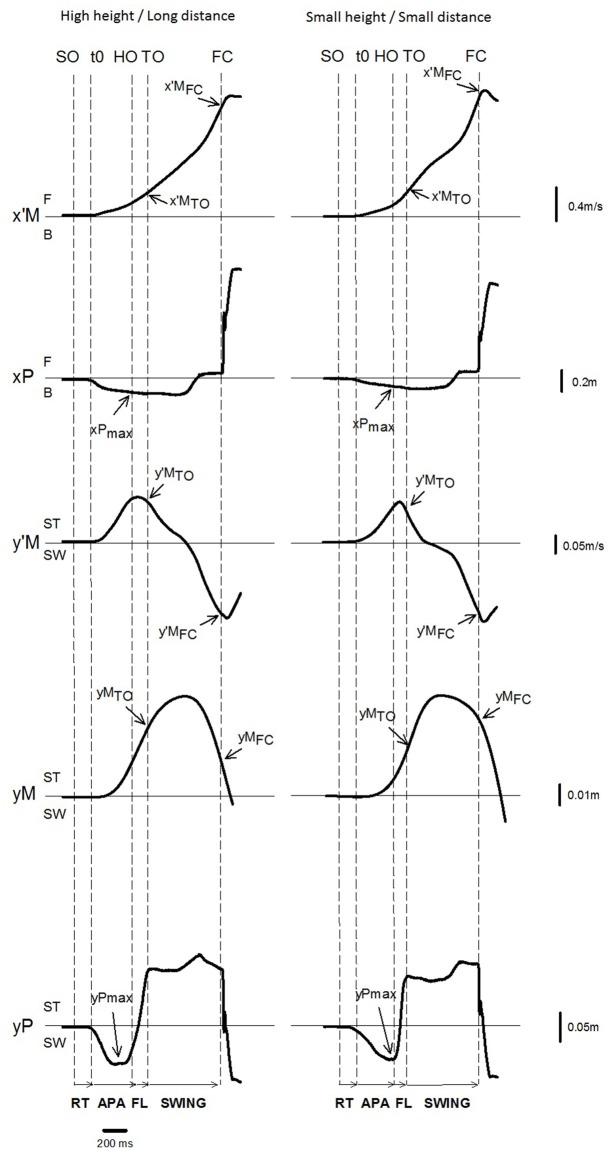
**Example of biomechanical traces and representation of the main experimental variables obtained for one representative subject initiating gait (one trial) in the reaction-time condition with the high height/long distance condition (left) and the small height/small distance condition (right).** *Anteroposterior (AP) direction* x′M: center of mass (COM) velocity; x′M_TO_, x′M_FC_: COM velocity at foot off and at foot contact. xP: center of pressure (COP) displacement; *x*Pmax: peak of COP displacement during APAs; F: forward; B: backward. *Mediolateral (ML) direction* y′M: ML COM velocity; y′M_TO_, y′M_FC:_ COM velocity at foot off and foot contact; yM: ML COM displacement; yM_FC_: COM displacement at foot contact; yP: ML COP displacement; yPmax: peak of COP displacement during APAs; and ST: stance limb; SW: swing limb. *Vertical* *dashed lines* SO: Go signal onset (in the reaction-time condition only); t_0_ onset variation of biomechanical traces; HO: swing heel off; FO: swing foot off; FC: swing foot contact. *Horizontal arrows*: RT: time-windows for reaction-time; APA: anticipatory postural adjustments FL: foot lift; SWING: swing phase.

Gait initiation was divided into three phases: APAs (from t_0_ to heel off), swing foot lift (from heel off to toe off) and swing phase (from toe off to foot contact, Figure 3). The duration of APAs along the ML and AP axes were computed separately, because the t_0_ times for these two axes did not necessarily occur simultaneously (Caderby et al., 2014). The amplitude of APAs was characterized by the peaks of the backward and lateral center of pressure shift obtained during the APAs time window. Center of mass velocity and displacement along the ML and AP axes were quantified at toe off and foot contact. The ML and AP center of mass position in the initial upright static posture was estimated by averaging the center of pressure position during the 250 ms period preceding the “all set” or the “go” signal, depending on the temporal pressure condition (Yiou et al., 2016). Spatiotemporal features of the swing phase of gait initiation that were investigated included: swing phase duration, AP center of mass velocity at foot contact, step length, step width, and dynamic stability at foot contact. Step length corresponded to the distance covered by the heel marker of the swing leg from the initial posture to foot contact. In addition, the vertical distance between the obstacle and the swing heel and swing toe markers was measured at the time when these markers passed over the obstacle. For each trial in the obstacle condition, the shorter of these two vertical distances was reported; this corresponded to the “foot clearance”. An adaptation of the “margin of stability” (MOS) introduced by Hof et al. (2005) was used to quantify ML dynamic stability at foot contact (thereafter referred to as “stability”). The MOS corresponded to the difference between the ML boundary of the base of support (BOS_ymax_) and the ML position of the “extrapolated center of mass” at swing foot contact (YcoM_FC_). Thus:

(7) $$\text{MOS}\;=\;{\text{BOS}}_{\text{ymax}}-{\text{YcoM}}_{\text{FC}}$$

Because kinematic data showed that participants first landed on the force plate with the swing heel or the swing toe, BOS_ymax_ was estimated using the ML position of the swing heel or metatarsal marker at foot contact. The ML distance between the position of the swing foot marker at foot contact (heel or toe) and the position of the stance metatarsal marker at t_0_ represented step width, and was representative of the size of the ML base of support. Based on the study by Hof et al. (2005) and the results from our previous studies (Caderby et al., 2014; Yiou et al., 2016), the ML position of the extrapolated center of mass at foot contact (YcoM_FC_) was calculated as follows:

(8) $${\text{YcoM}}_{\text{FC}}\;=\;yM_{\text{FC}}+\frac{y^{\prime }M_{\text{FC}}}{\omega_{0}}$$

where *yM_FC_* and y’M_FC_ are respectively the ML center of mass position and velocity at foot contact, and *ω*_0_ is the eigen frequency of the body, modeled as an inverted pendulum and calculated as follows:

(9) $$\omega_{0}\;=\;\sqrt{\frac{g}{l}}$$

where *g* = 9.81 m/s^2^ is the gravitational acceleration and *l* is the length of the inverted pendulum, which in this study correspond to 57.5% of body height (Winter et al., 1990).

ML dynamic stability at foot contact is preserved on the condition that YcoM_FC_ is within BOS_ymax_, which corresponds to a positive MOS. A negative MOS indicates a ML instability and implies that a corrective action (e.g., in the form of an additional lateral step) is required to maintain balance.

### Theoretical Variables

In order to test the validity of the model, the theoretical ML position and velocity of the center of mass at foot contact were computed by implementing the model with the initial center of mass set (ML center of mass position and velocity at foot off) and the swing phase duration obtained in each of the experimental trials. This gave theoretical values which were then compared with the experimental ones. The ML APAs were found to be scaled with swing duration in the experimental conditions (see “Results” Section); thus, these theoretical values are referred to the “theoretical conditions with APAs scaling”. The model was then used to assess whether postural stability at foot contact would be degraded if the ML APAs were not scaled to swing duration, i.e., if there was no adaptation of the initial center of mass set to the obstacle height and distance. For this purpose, the theoretical ML position and velocity of the center of mass at foot contact were again computed, but this time by implementing the model with: (i) the mean ML position and velocity of the center of mass at foot off obtained for each subject in the reaction-time and self-initiated obstacle-free (control) conditions; and (ii) the swing phase duration obtained in each experimental trial. The theoretical extrapolated center of mass position and the theoretical MOS at foot contact were then calculated by following the same procedure used for the experimental data. The theoretical values obtained with this procedure are referred to as the “theoretical conditions without APA scaling”.

### Statistics

Mean values and standard deviations were calculated for each variable in the experimental and theoretical conditions. The normality of data was checked using the Kolmogorov-Smirnov test and the homogeneity of variances was checked using the Bartlett test. To test the influence of obstacle height, obstacle distance and temporal pressure, a [3 (“obstacle height”: 2.5%, 5% and 10% of the subject’s height) × 3 (“obstacle distance”: 10%, 20% and 30% of the subject’s height) × 2 (“temporal pressure”: reaction-time and self-initiated)] ANOVA with repeated measures was used on each experimental variable.

To test the validity of the model, a [3 (“obstacle height”) × 3 (“obstacle distance”) × 2 (“temporal pressure”) × 2 (“modeling with APAs scaling”: experimental conditions vs. theoretical conditions with APA scaling)] was used on the following variables: ML center of mass position and velocity, ML extrapolated center of mass, and MOS at foot contact. Linear correlations between these experimental and theoretical variables were calculated using Pearson’s correlation coefficient. Finally, to test the effect of APA scaling on postural stability, a [3 (“obstacle height”) × 3 (“obstacle distance”) × 2 (“Modeling without APA scaling”: experimental conditions vs. theoretical conditions with no APA scaling)] RM ANOVA was used on the following variables: ML center of mass position and velocity, ML extrapolated center of mass, and MOS at foot contact. The alpha level was set at 0.05. A Tukey *post hoc* test was used when necessary.

## Results

### Description of the Biomechanical Traces in the Experimental Conditions

The time course of the biomechanical traces was globally similar in the different temporal and obstacle conditions. The traces obtained in two representative conditions are reported in Figure 3. Swing heel off was systematically preceded by postural dynamics that corresponded to APAs. During these APAs, the center of pressure displacement reached a peak value in a backward direction (see the negative variation of the xP trace in Figure 3) and towards the swing leg side (negative variation of the yP trace), while the center of mass velocity was directed forwards (positive variation of the x’M trace) and towards the stance leg side (positive variation of the y’M trace). The ML center of mass velocity trace reached a first peak value towards the stance leg side at around heel off. This trace, then fell towards the swing leg side and a second peak value towards this side was reached a few milliseconds after foot contact. The ML center of mass shift trace was bell-shaped and reached a peak value toward the stance leg side at the beginning of the swing phase. The AP center of mass velocity increased progressively until it reached a peak value a few milliseconds after swing foot contact, while the center of mass was continuously shifted forward. Differences across the conditions are reported in the paragraphs below.

### Stability

#### Height Effect

**Figure 4:**
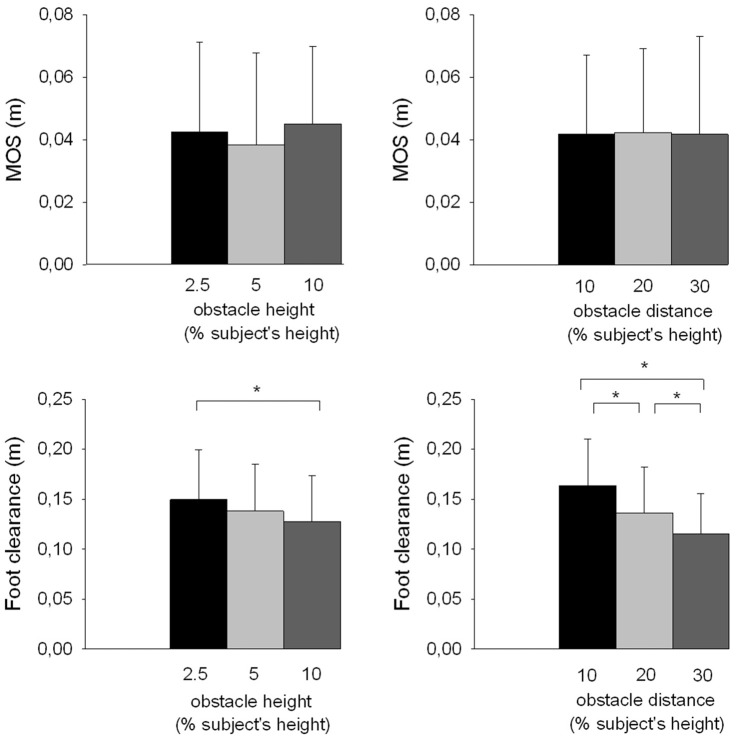
**Effects of obstacle height and distance on stability parameters.** Reported are mean values (all participants together) ± 1 SD. MOS, margin of stability. *Indicates a significant difference between bars.

Stability can be evaluated from foot clearance and the MOS. The risk of the swing foot striking the obstacle, which might then endanger balance, increased as foot clearance decreased. The MOS is used to quantify ML dynamic stability at foot contact. The results showed that the foot clearance significantly decreased with obstacle height (*F*_(2,26)_ = 9.25, *p* < 0.001; Figure 4). In contrast, there was no significant effect of the obstacle height on the MOS value (*F*_(2,26)_ = 2.57, *p* > 0.05) and related center of mass components, i.e., the ML center of mass shift (*F*_(2,26)_ = 0.31, *p* > 0.05) and velocity (*F*_(2,26)_ = 0.46, *p* > 0.05), and extrapolated center of mass position at foot contact (*F*_(2,26)_ = 1.95, *p* > 0.05).

#### Distance Effect

The results showed that foot clearance also significantly decreased with obstacle distance (*F*_(2,26)_ = 30.07, *p* < 0.001; Figure 4). There was no effect of the obstacle distance on the MOS (*F*_(2,26)_ = 0.01, *p* > 0.05), the ML center of mass shift (*F*_(2,26)_ = 0.99, *p* > 0.05) and velocity (*F*_(2,26)_ = 0.64, *p* > 0.05), and the extrapolated center of mass position at foot contact (*F*_(2,26)_ = 0.11, *p* > 0.05).

#### Temporal Pressure Effect

There was no significant effect of the temporal pressure on the following variables: foot clearance (*F*_(1,13)_ = 3.77, *p* > 0.05), MOS value (*F*_(1,13)_ = 0.96, *p* > 0.05), ML center of mass shift (*F*_(1,13)_ = 0.95, *p* > 0.05) and velocity (*F*_(1,13)_ = 0.55, *p* > 0.05) and extrapolated center of mass position at foot contact (*F*_(1,13)_ = 0.35, *p* > 0.05).

### Postural and Foot Lift Phase

#### Height Effect

**Figure 5:**
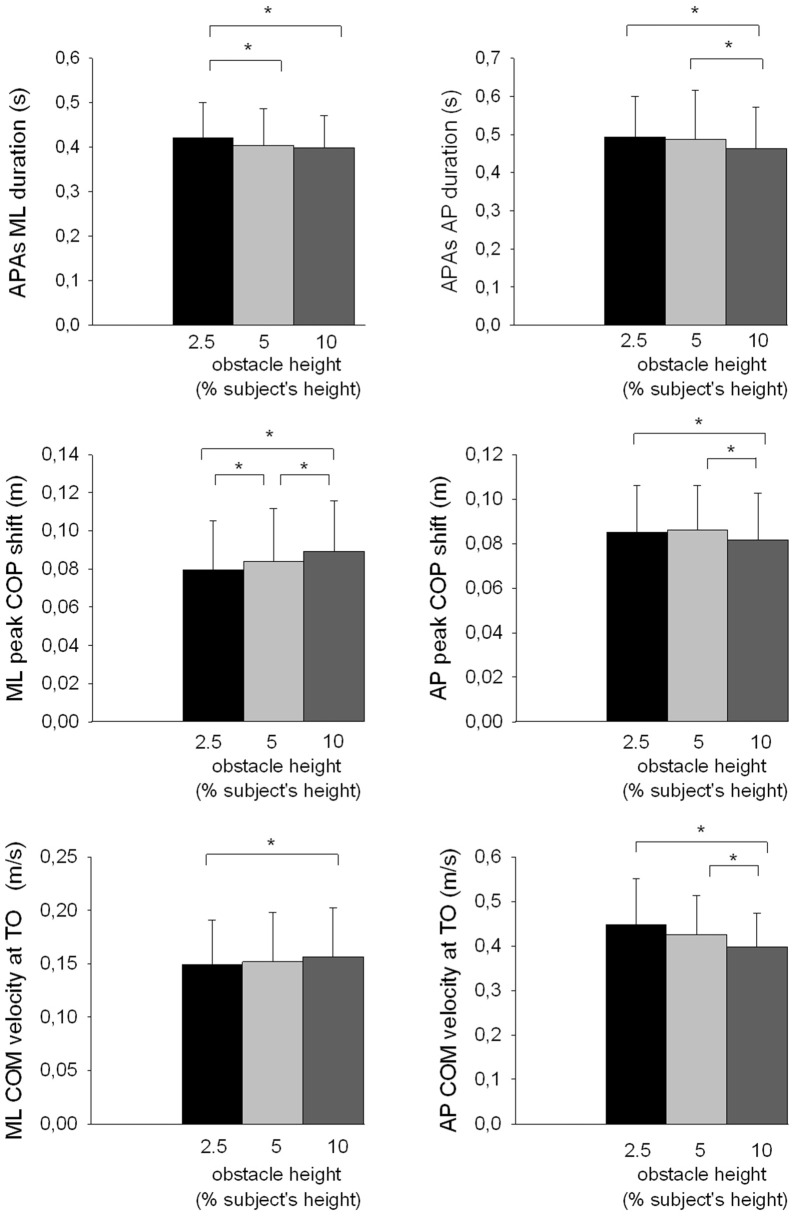
**Effects of obstacle height on selected ML and AP postural parameters.** Reported are mean values (all participants together) ± 1 SD. APAs, anticipatory postural adjustments; TO, toe off; COP, center of pressure; COM, center of mass. *Indicates a significant difference between bars.

The results showed that there was a significant effect of the obstacle height on the duration of APAs along the ML axis (*F*_(2,26)_ = 5.63, *p* < 0.01, Figure 5) and the AP axis (*F*_(2,26)_ = 9.38, *p* < 0.001), and on the duration of the foot-lift phase (*F*_(2,26)_ = 6.18, *p* < 0.01). Each of these temporal variables decreased when the obstacle height increased. With regard to the spatial variables, results showed that both the peak of anticipatory ML center of pressure shift (*F*_(2,26)_ = 21.44, *p* < 0.001) and the ML center of mass velocity at toe off (*F*_(2,26)_ = 4.36, *p* < 0.05) significantly increased with obstacle height. In contrast, the peak of anticipatory backward center of pressure shift (*F*_(2,26)_ = 11.43, *p* < 0.001), the differential between the center of pressure and the center of mass position *F*_(2,26)_ = 8.15, *p* < 0.01) and the forward center of mass velocity at toe off (*F*_(2,26)_ = 13.86, *p* < 0.001) significantly decreased with obstacle height. The obstacle height constraint therefore had a similar effect on the temporal component of APAs along the AP and ML axes, but had an opposite effect on the spatial component of APAs along these two axes.

#### Distance Effect

Increasing the obstacle distance had a very different effect on the postural and foot lift phases compared with increasing the obstacle height. Indeed, the results showed that obstacle distance had no significant effect on the duration of APAs along the ML axis (*F*_(2,26)_ = 1.44, *p* > 0.05). However, it did have a significant effect on the APA duration along the AP axis (*F*_(2,26)_ = 3.77, *p* < 0.05) and on the duration of the foot lift (*F*_(2,26)_ = 21.53, *p* < 0.001). Specifically, these two variables increased with obstacle distance. The results further showed that there was no significant effect of the obstacle distance on the following spatial variables: peak of anticipatory ML (*F*_(2,26)_ = 0.62, *p* > 0.05) and AP center of pressure shift (*F*_(2,26)_ = 1.00, *p* > 0.05), ML (*F*_(2,26)_ = 1.78, *p* > 0.05) and AP (*F*_(2,26)_ = 2.18, *p* > 0.05) center of mass shift at toe off, and ML center of mass velocity at toe off (*F*_(2,26)_ = 1.30, *p* > 0.05). In contrast, the forward center of mass velocity at toe off increased significantly with obstacle distance (*F*_(2,26)_ = 30.51, *p* < 0.001).

#### Temporal Pressure Effect

The results showed that the following temporal variables were significantly shorter in the reaction-time block than in the self-initiated block: duration of APAs along the AP axis (*F*_(1,13)_ = 61.63, *p* < 0.001) and ML axis (*F*_(1,13)_ = 31.6, *p* < 0.001), and duration of foot lift (*F*_(1,13)_ = 16.99, *p* < 0.01). The following spatial variables reached a significantly larger value in the reaction-time block than in the self-initiated block: peak of anticipatory ML (*F*_(1,13)_ = 20.04, *p* < 0.001) and AP (*F*_(1,13)_ = 41.82, *p* < 0.001) center of pressure shift, and ML center of mass velocity at foot off (*F*_(1,13)_ = 11.60, *p* < 0.01). In contrast, the ML (*F*_(1,13)_ = 1.98, *p* > 0.05) and AP (*F*_(1,13)_ = 2.45, *p* > 0.05) shift of the center of mass at foot off were not significantly different for the two temporal pressure blocks.

### Swing Phase

#### Height Effect

The results showed that the duration of the swing phase significantly increased with obstacle height (*F*_(2,26)_ = 58.07, *p* < 0.001). In contrast, there was no significant effect of the obstacle height on the step length (*F*_(2,26)_ = 2.77, *p* > 0.05), step width (*F*_(2,26)_ = 0.59, *p* > 0.05) and motor performance (in terms of forward center of mass velocity at swing foot contact; *F*_(2,26)_ = 0.74, *p* > 0.05). Finally, the results showed that there was no change in swing foot strike patterns with changes to obstacle height; here, subjects landed on the force plate with the heel first in 85% of the trials.

#### Distance Effect

The results showed that there was no effect of the obstacle distance on the duration of the swing phase (*F*_(2,26)_ = 2.57, *p* > 0.05) and step width *F*_(2,26)_ = 0.05, *p* > 0.05). In contrast, there was a significant effect of the obstacle distance on the step length (*F*_(2,26)_ = 23.05, *p* < 0.001) and motor performance (*F*_(2,26)_ = 6.72, *p* < 0.01). Both variables increased with distance. Finally, the results showed that there was a significant effect of obstacle distance on the foot strike pattern, with the ratio of forefoot strike increasing with obstacle distance (*F*_(2,26)_ = 7.37, *p* < 0.01). This ratio increased from 6.7% for the small distance obstacle condition to 20.7% for the long distance obstacle condition.

#### Temporal Pressure Effect

The results showed that there was a significant effect of the temporal pressure on the duration of the swing phase (*F*_(1,13)_ = 10.81, *p* < 0.01). This duration was longer in the reaction-time block than in the self-initiated block. In contrast, there was no effect of the temporal pressure on the following variables: step length (*F*_(1,13)_ = 0.58, *p* > 0.05), step width (*F*_(1,13)_ = 0.20, *p* > 0.05), motor performance (*F*_(1,13)_ = 0.04, *p* > 0.05) and foot strike pattern (the mean percentage of the heel-strike pattern was 86%).

### Validation of the Mechanical Model

**Figure 6:**
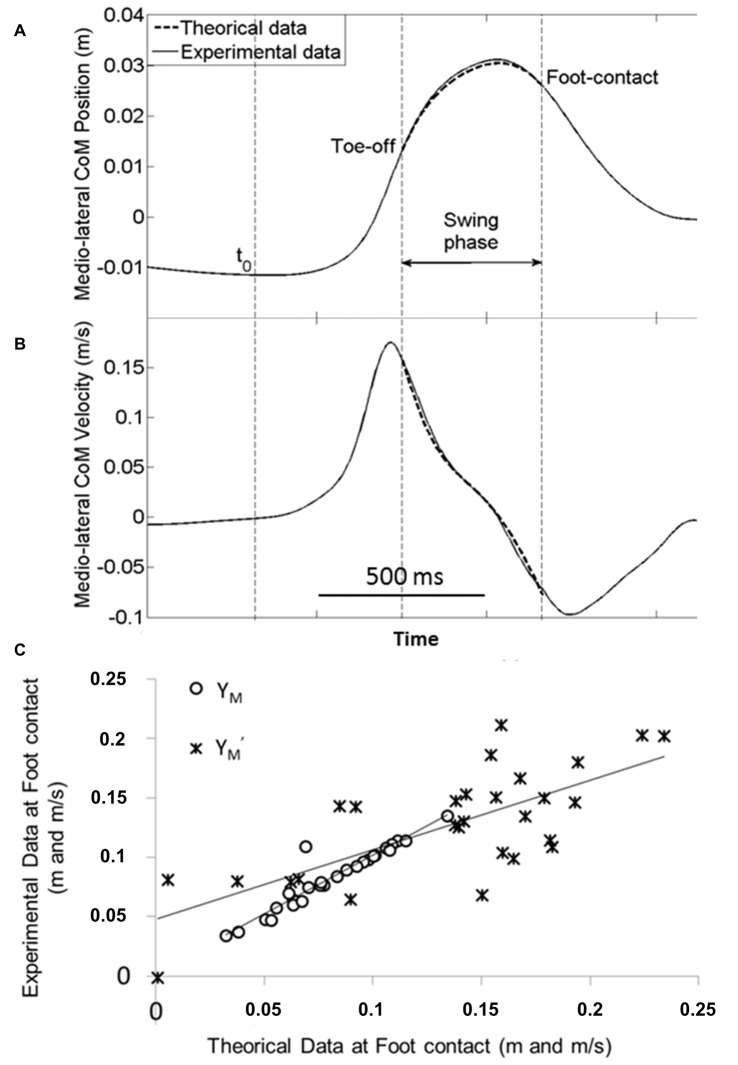
**Validation of the mechanical model.** Typical experimental (full line) and theoretical (dash line) time-course traces of the ML center of mass shift **(A)** and velocity **(B)** are superimposed during the swing phase (from swing foot off to foot contact). Traces are obtained from one representative subject in the reaction-time condition with the medium height and short distance obstacle. **(C)** Example of linear regression between experimental vs. theoretical data obtained in the obstacle free (control) condition. Each point represents the average value of the ML center of mass position (Y_M_) and velocity (Y′_M_) at foot contact in the control conditions (self-initiated and reaction-time conditions pooled together) for each of the 14 subjects. Note the excellent fit between the experimental and theoretical data.

A visual analysis of Figure 6 illustrates the excellent fit between the experimental traces and those obtained with the mechanical model. The best fit between experimental (dashed line) and theoretical (full line) data was obtained for a stiffness of the hip abductor muscles of about 1000 N/m. This value corroborates with previous data in the literature (Morasso and Schieppati, 1999). This corresponds to a restoring force of approximately *T* = 50 N, applied at the center of mass. This close fit was further strengthened by the finding that there was no significant effect of the factor “modeling with APA scaling” on the MOS and on the related center of mass components. In addition, there was no interaction between this factor and obstacle height, obstacle distance and temporal pressure for any of these variables. In contrast, there was a significant positive correlation between the theoretical data (obtained in the conditions with ML APA scaling) and the experimental data for the MOS (*r* = 0.42, *p* < 0.05), the ML center of mass position (*r* = 0.94, *p* < 0.001) and the ML center of mass velocity (*r* = 0.72, *p* < 0.001) at foot contact. Collectively, these results validate the mechanical model.

### Comparison of Experimental Data and Theoretical Data Obtained in the Conditions Without Mediolateral APA Scaling

**Figure 7:**
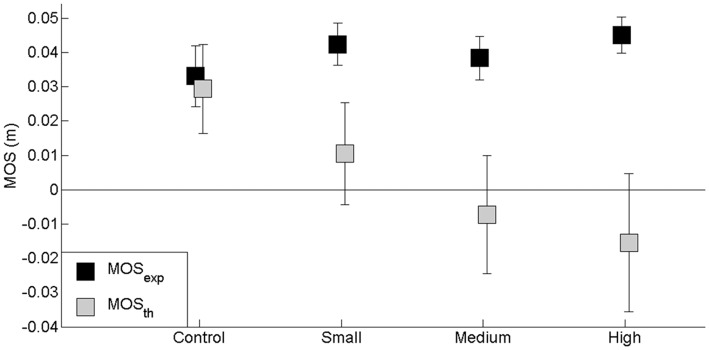
**Effects of obstacle height on the experimental (MOS_exp_) and theoretical (MOS_th_) “margin of stability” (MOS) values computed in the conditions with no APA scaling.** The height of the obstacle is indicated in the abscissa (small, medium and high). In the control condition, there was no obstacle. Reported are mean values (all participants together) ± 1 SD. Note that the experimental MOS values remained unchanged, while theoretical MOS values decreased with obstacle height.

In the theoretical conditions without ML APA scaling, the same initial ML center of mass set used in the control condition (obstacle-free condition) was introduced into the conditions where an obstacle had to be cleared (see “Materials and Methods” Section). The results showed there was a significant effect of the factor “modeling without APA scaling” on the MOS (*F*_(2,26)_ = 4.77, *p* < 0.05) and with the exception of the ML center of mass position at foot contact (*F*_(2,26)_ = 1.63, *p* > 0.05), on each of the MOS-related center of mass components, i.e., peak of ML velocity (*F*_(2,26)_ = 8.73, *p* < 0.001) and extrapolated center of mass (*F*_(2,26)_ = 4.84, *p* < 0.05) at foot contact. Specifically, the mean MOS value was significantly lower in the theoretical conditions compared with the experimental conditions, and the extrapolated center of mass reached positions closer to the lateral boundary of the base of support. In addition, the peak of ML center of mass velocity at foot contact—which was directed towards the swing leg side—reached a greater value in the theoretical conditions than in the experimental conditions. Also, there was a “modeling without APA scaling” × “obstacle height” interaction on the MOS (*F*_(2,26)_ = 4.77, *p* < 0.05) and on each related variable. Most interestingly, the difference in the MOS value between the experimental and theoretical conditions without APA scaling increased progressively when the obstacle height increased (Figure 7). A negative MOS value was even reached for the middle height obstacle. Finally, the results showed that there was no significant “modeling without APA scaling” × “obstacle distance” interaction (*F*_(2,26)_ = 0.22, *p* > 0.05) or “modeling without APA scaling” × “temporal pressure” interaction (*F*_(2,26)_ = 0.16, *p* > 0.05). These results thus illustrate how postural stability can be expected to degrade in cases where ML APAs are not scaled according to swing duration.

## Discussion

The goal of the present study was to investigate how the CNS controls postural stability during gait initiation when negotiating obstacles of different heights and distances under low and high temporal pressure constraints. Based on a mechanical model of the body falling laterally under the influence of gravity and submitted to an elastic restoring force, the functional link between the observed ML APA scaling and the maintenance of postural stability across the experimental conditions was first discussed. This was followed by a discussion of the way in which the AP and ML components of APAs need to be coordinated to ensure safe body progression. Globally, the results illustrate the capacity of the CNS to adapt coordination between the postural and focal components of a motor task to meet various spatial and temporal constraints.

### Scaling Mediolateral APAs to Swing Duration Allows the Maintenance of Postural Stability

As expected, increasing the obstacle height resulted in a significant increase in swing duration (Chou et al., 2001; Hahn and Chou, 2004), thereby mechanically increasing the potential for lateral imbalance during the swing phase of gait initiation. Indeed, previous studies have reported that the swing phase of gait initiation could be assimilated to a ML (Lyon and Day, 1997, 2005) and forward (Lepers and Brenière, 1995) ballistic center of mass fall around the stance ankle, with gravity being the main motor of action. Increasing the duration of this phase may therefore theoretically lead to a larger center of mass motion and velocity at the end of this ballistic phase, i.e., at foot contact. The original model of the whole body falling towards the swing leg side after swing foot off, which was developed in the present study, is in accordance with this statement. Indeed, when the initial center of mass set (i.e., center of mass shift and velocity at foot off) remained the same as in the obstacle-free condition, it was found that artificially increasing the swing phase duration directly impacted on the center of mass set at foot contact. As a consequence, postural stability at this instant was degraded, as revealed by a decrease in the theoretical MOS values. In contrast to this effect of obstacle height, increasing the obstacle distance did not result in any significant change in the swing phase duration. This is coherent with the finding that both step length and step velocity increased with obstacle distance. Thus, as expected, the theoretical center of mass set at foot contact and the related degree of postural stability remained unchanged when this distance increased.

Although the theoretical model revealed the potential for increased instability with obstacle height, the results obtained in the experimental conditions showed that there was no main effect of obstacle height on the center of mass set at foot contact and on the related level of stability. Similar findings were observed in recent studies (Chou et al., 2001; Hahn and Chou, 2004) that examined the clearance of obstacles of varying height during steady walking. This led the authors to suggest the existence of some forms of adaptive postural mechanisms, although these mechanisms were not described. The present results show that the anticipatory peak of ML center of pressure shift increased along with obstacle height. This increase is responsible for a subsequent increase in the ML center of mass velocity at toe off. As stated above, it is clear from the theoretical model that if such an increase in the initial center of mass set had not occurred when the obstacle height increased, a lower state of postural stability would have been reached at foot contact, as shown by the lower theoretical MOS values. Thus, in order to maintain an equivalent stability in the experimental conditions, the additive strategy would be needed to compensate for insufficient APAs, e.g., in the form of lateral stepping so as to increase the base of support width (Zettel et al., 2002; Caderby et al., 2014). If this is still insufficient, because not enough time is available to position the swing foot laterally, a strategy of lateral leg crossover stepping, linked with a high risk of lateral falling (Patton et al., 2006), may be necessary to recover balance. Our results thus show that the CNS precisely scales the ML APAs to the duration of the swing phase, so as to maintain an equivalent postural stability at foot contact across the experimental conditions. The finding that obstacle distance had no influence on ML APAs parameters and the related initial center of mass set (in contrast with obstacle height) is in line with this statement, because swing duration did not vary with this obstacle feature.

The present findings are in accordance with the notion that postural stability at the end of a voluntary leg movement is a major parameter taken into account during the programming of APAs (Do et al., 1991; Nouillot et al., 2000). Do et al. (1991) used a lower limb flexion-extension executed as fast as possible to test the influence of final stability on APAs. The initial body posture was bipedal, while the final one was either bipedal (stable posture) or unipedal (unstable posture). The biomechanical and electromyographic data showed that ML APAs were larger when the final posture was unstable, because of the need to propel the center of mass further (i.e., above the stance foot) to maintain stability in the final posture. Similarly, the amplitude of the ML APAs in the present study increased along with the potential for instability at foot contact, which corresponded to the end of the gait initiation process. In contrast to the study by Do et al. (1991), the potential for increased instability at the end of gait initiation was masked in our study, because the MOS remained the same across the experimental conditions. A similar remark can be made with regard to previous studies which focused on the effect of various environmental (Chou et al., 2001; Hahn and Chou, 2004; Yiou et al., 2011, 2016) or temporal constraints (Yiou et al., 2012b; Hussein et al., 2013; Caderby et al., 2014) on the control of ML stability during dynamic tasks (e.g., leg flexion, gait initiation and steady walking). In the present study, this potential for instability was revealed in the theoretical trials, where it was found that without APA scaling the MOS values decreased when obstacle height was increased. The present results thus demonstrate the imperative need to adequately scale the ML APAs features to the swing phase duration in order to maintain an optimal stability. Moreover, the invariance of the MOS value across the experimental conditions, despite the presence of potential instability, adds to the growing evidence that this parameter may function as a balance control parameter, as previously suggested in the literature (e.g., Yiou et al., 2011, 2012b; Caderby et al., 2014; Nakano et al., 2016).

This invariance implies that the CNS is able to precisely predict the potential instability elicited by obstacle clearance and that it scales the spatiotemporal parameters of the ML APAs accordingly. The results of this study are thus in accordance with the view that in programming APAs, the CNS uses internal models that takes into account the dynamic consequences of an expected perturbation and generates responses to counter their effect (e.g., Flanagan and Wing, 1997; Wing et al., 1997). More specifically, internal models would be used to predict the effect of the gravitational forces acting on the whole-body during obstacle clearance. This prediction would serve to program, adaptive APAs so as to maintain unchanged stability despite the variations in obstacle constraints. The notion that internal models integrate external forces (such as gravity or Coriolis forces) acting on body segments to plan and execute movements, has been classically proposed for various voluntary upper limb movements, such as grip force with load during object manipulation (e.g., Johansson and Cole, 1992; Flanagan and Wing, 1997; Kawato, 1999; Wolpert and Flanagan, 2001), arm movement in the vertical plane (e.g., Papaxanthis et al., 2005; Gaveau and Papaxanthis, 2011), arm reaching (Cohn et al., 2000) etc. The results of the current study further suggest that such internal models of gravity may also be used to plan and execute the postural component of a whole body motor task.

### Coordination Between Mediolateral and Anteroposterior Components of APAs Allows Safe Body Progression

Surprisingly, the results also showed that the duration of both the ML APAs and the foot lift phase decreased with obstacle height. Less time was therefore allocated by participants to propelling the center of mass laterally before triggering the ballistic phase of gait initiation. This reduction in time may seem at odds with the need to increase the ML center of mass set at foot off, as argued above. We propose that it is linked to the spatial constraints exerted on the progression velocity in the AP direction. It can indeed reasonably be speculated that delaying the time of swing foot off in the presence of a high obstacle would increase the forward fall of the center of mass (allowing participants to get closer to the obstacle), as well as the amplitude of the forward center of mass velocity at foot off (Lepers and Brenière, 1995). By so doing, less time would then be allocated for clearing the obstacle with the trailing leg during the following swing phase, with a consequent increased risk of tripping over the obstacle. Instead, the results showed that the forward center of mass shift and velocity at foot off both decreased with obstacle height, which might be a combined effect of this shortened delay for swing foot off with the reduced amplitude of the anticipatory backward center of pressure shift. The amplitude of AP and ML APAs were thus both scaled according to swing duration but in an opposite way. Note that a similar strategy for AP APAs attenuation has already been reported in a study that compared stepping over an obstacle in reaction to rapid surface translation with stepping when there is no obstacle to be cleared (Zettel et al., 2002). The reduction in time taken to lift the swing foot might reflect a protective strategy directed to clear the obstacle safely by reducing the chances of contact between the trailing leg and the obstacle. It is however noteworthy that the vertical distance between the swing foot and the top of the obstacle at the time of obstacle clearance (i.e., the “foot clearance”) decreased with obstacle height. In our trials, we did not observe any obstacle contact; thus, we believe that the obstacle (height and distance) and the velocity constraints of the present study were not putting young, healthy participants at risk of forward tripping.

Given the precise scaling of the initial ML center of mass set required to maintain stability across the obstacle conditions (see Figure 7), the present findings suggest that the CNS must necessarily have taken into account the reduction in time allocated to lift the swing foot to program the ML APAs’ amplitude. Part of the observed increase in the peak of anticipatory ML center of pressure shift may therefore serve to compensate for this shortened duration so that an adequate initial ML center of mass set can be reached to maintain stability. In other words, it is likely that constraints imposed on the progression direction (which are likely responsible for the reduction in time allocated for swing toe off, as argued above) were integrated into the programming of APAs in the ML direction. This statement adds to the growing evidence that the CNS exerts a global control over the anticipatory postural dynamics in the horizontal plane (Caderby et al., 2014) rather than an independent control of APAs along the AP and ML axes and on the associated postural function (forward body progression and ML stability, respectively). This coordination between the ML and AP components of APAs thus seems to be an imperative condition for both safely clearing the obstacle and reaching a stable state at foot contact. Thus, in addition to the need to coordinate each postural component of the task (the AP and the ML postural components) with the focal one, the CNS also needs to coordinate the postural components between them so that participants can safely clear the obstacle.

### Temporal Pressure-Induced Adaptive Changes of Mediolateral and Anteroposterior APAs

Compensation for a reduced ML APAs duration by an increase in the ML APAs amplitude was found in the present study when comparing the high and low temporal pressure conditions. Specifically, the duration of APAs in the high pressure condition was shorter and the peaks of anticipatory ML and AP center of pressure shift were larger than in the low pressure condition. A similar effect of temporal pressure was previously reported in the literature for various stepping tasks such as gait initiation with or without an obstacle to clear (Yiou et al., 2016), or rapid leg flexion (Yiou et al., 2012b; Hussein et al., 2013). It is presumed that these changes in the spatiotemporal APAs parameters under high temporal pressure reflect a strategy to hasten the onset of the voluntary movement (swing foot off) so as to meet the instruction to initiate the step as soon as possible after the GO signal, while maintaining the same stability and progression velocity. Obstacle height and temporal pressure, thus induced similar adaptive changes in the ML APAs parameters. This similitude could be explained by the fact that increasing the obstacle height and the temporal pressure level both required an earlier swing foot off and induced a longer swing phase duration. For this reason, combining these two constraints in one single condition (i.e., clearing a high obstacle within a high temporal pressure) may have been particularly challenging for the postural control system. The fact that postural stability was maintained in such a challenging condition further reveals the adaptability of this system.

The present results may be discussed in regards to recent studies which focused on the effect of temporal pressure on ML stability during ongoing walking with the goal to cross an obstacle (e.g., Moraes et al., 2007; Nakano et al., 2015, 2016). In these studies, participants avoided a virtual planar obstacle that could suddenly appear one step before the obstacle crossing, thus inducing a temporal pressure. In the condition without temporal pressure (control condition), the obstacle could be seen by participants when they stood in their initial posture. Under temporal pressure, the authors found that the extrapolated center of mass position at the swing foot contact was located further toward the swing leg side as compared to the control condition. The MOS however, remained unchanged because of a greater lateral step placement. In the present study, no such effect of temporal pressure on the actual and extrapolated center of mass position or on foot placement was observed. The MOS and the related center of mass components remained, however the same as in the low temporal pressure condition. This invariance was due to the above reported changes in APAs parameters with temporal pressure. These discrepancies between the present study and the literature might possibly be ascribed to the time allocated to plan an efficient anticipatory strategy to maintain postural stability. In the present study, participants could indeed visually catch the features of the obstacle largely before the imperative “go” signal in the high pressure condition. In other words, they had plenty of time to predict the postural disturbance associated with the forthcoming task, and they could thus plan the APAs parameters accordingly. In line, MacKinnon et al. (2007) reported that the spatiotemporal features of APAs for gait initiation were progressively assembled before the deliverance of the “go” signal. In contrast, in the above reported studies, the obstacle appeared just one step before it had to be cleared. Participants had therefore much less time than in the present study to plan in advance the level of anticipatory postural dynamics required to maintain stability at foot-contact. Such a situation may potentially be detrimental to stability since it is known that vision is used in a feedforward rather than in on-line mode to regulate obstacle clearance during ongoing locomotion (Patla and Vickers, 1997). To maintain stability, participants in these studies thus needed to use an additive strategy of lateral foot placement to maintain stability. Future studies will investigate this hypothesis by enabling participants to catch the obstacle features with various delays before and after the deliverance of the “go” signal.

## Conclusion

The results of this study show that the CNS is able to scale and coordinate the ML and AP components of APAs according to obstacle constraints and related variations in swing duration. This capacity allows participants to safely clear the obstacle and maintain optimal postural stability. These results were strengthened by the findings obtained with the mechanical model, which revealed how stability would be degraded if the ML APAs were not scaled to swing duration. These findings imply that the CNS is able to precisely predict the potential instability elicited by obstacle clearance and that it scales the spatiotemporal parameters of APAs according to this prediction. The results offer a better understanding of how the body adapts to environmental constraints in order to ensure safe and efficient whole-body progression. In a future study, we will investigate the strategies of young, healthy adults and compare them with those adopted by older adults (fallers and non-fallers) in the maintenance of stability in a similarly complex environment.

## Author Contributions

EY, RA, CT, OL, PF: designed the study; collected, analyzed and interpreted the data; drafted and revised the manuscript; gave final approval.

## Funding

This research was funded by the French Government.

## Conflict of Interest Statement

The authors declare that the research was conducted in the absence of any commercial or financial relationships that could be construed as a potential conflict of interest.
